# Sensitivity and Specificity of Rapid SARS-CoV-2 Antigen Detection Using Different Sampling Methods: A Clinical Unicentral Study

**DOI:** 10.3390/ijerph19116836

**Published:** 2022-06-02

**Authors:** Faisal Alonaizan, Jehan AlHumaid, Reem AlJindan, Sumit Bedi, Heba Dardas, Dalia Abdulfattah, Hanadi Ashour, Mohammed AlShahrani, Omar Omar

**Affiliations:** 1 Department of Restorative Dental Sciences, College of Dentistry, Imam Abdulrahman Bin Faisal University, Dammam 34212, Saudi Arabia; falonaizan@iau.edu.sa; 2 Department of Preventive Dental Sciences, College of Dentistry, Imam Abdulrahman Bin Faisal University, Dammam 34212, Saudi Arabia; jaalhumaid@iau.edu.sa (J.A.); sbrajinder@iau.edu.sa (S.B.); 3 Department of Microbiology, College of Medicine, Imam Abdulrahman Bin Faisal University, Dammam 34212, Saudi Arabia; raljindan@iau.edu.sa; 4 Emergency Department, King Fahad University Hospital, Al Khobar 34445, Saudi Arabia; hadardas@iau.edu.sa; 5 Clinical Nursing Supervisor Operating Room, King Fahad University Hospital, Al Khobar 34445, Saudi Arabia; dabdulfattah@iau.edu.sa; 6 College of Dentistry, Imam Abdulrahman Bin Faisal University, Dammam 34212, Saudi Arabia; hmashour@iau.edu.sa; 7 Department of Emergency Medicine, College of Medicine, Imam Abdulrahman Bin Faisal University, Dammam 34212, Saudi Arabia; msshahrani@iau.edu.sa; 8 Department of Biomedical Dental Sciences, College of Dentistry, Imam Abdulrahman Bin Faisal University, Dammam 34212, Saudi Arabia

**Keywords:** coronavirus/SARS-CoV-2, diagnostic systems, fluorescent immunoassay, oropharyngeal swab, nasal swab, saliva swab

## Abstract

Rapid antigen detection of SARS-CoV-2 has been widely used. However, there is no consensus on the best sampling method. This study aimed to determine the level of agreement between SARS-CoV-2 fluorescent detection and a real-time reverse-transcriptase polymerase chain reaction (rRT-PCR), using different swab methods. Fifty COVID-19 and twenty-six healthy patients were confirmed via rRT-PCR, and each patient was sampled via four swab methods: oropharyngeal (O), nasal (N), spit saliva (S), and combined O/N/S swabs. Each swab was analyzed using an immunofluorescent Quidel system. The combined O/N/S swab provided the highest sensitivity (86%; Kappa = 0.8), followed by nasal (76%; Kappa = 0.68), whereas the saliva revealed the lowest sensitivity (66%; kappa = 0.57). Further, when considering positive detection in any of the O, N, and S samples, excellent agreements with rRT-PCR were achieved (Kappa = 0.91 and 0.97, respectively). Finally, among multiple factors, only patient age revealed a significant negative association with antigenic detection in the saliva. It is concluded that immunofluorescent detection of SARS-CoV-2 antigen is a reliable method for rapid diagnosis under circumstances where at least two swabs, one nasal and one oropharyngeal, are analyzed. Alternatively, a single combined O/N/S swab would improve the sensitivity in contrast to each site swabbed alone.

## 1. Introduction

The coronavirus disease of 2019 (COVID-19) continues to pose challenges that require efficient methods, such as rapid and precise testing techniques, to limit its spread. Rapid antigen detection (RAD) systems have been proven to have relatively high sensitivity in the detection of SARS-CoV-2 (severe acute respiratory syndrome coronavirus 2) infection, in parallel with being more economical and time efficient when compared to RT-PCR [1,2]. However, RAD tests are considered inferior to RT-PCR in terms of sensitivity, which may be related to the method used for sampling [3].

For large-scale testing to become a reality, innovation in sampling techniques has been suggested. This requires the consideration of different sampling methods, including the choice of swab type and the sampling location. The different recommended swabbing techniques include nasopharyngeal swab (NP), nasal mid-turbinate swab (N), anterior nares nasal swab (AN), oropharyngeal swab (O), or a saliva specimen (S) [4]. Amongst these, NP sampling with flocked swabs has been the most accepted technique for SARS-CoV-2 testing, due to its high diagnostic yield, as evidenced by the U.S. Food and Drug Administration (FDA) [5]. However, NP swabs are known to be inconvenient for patients and require samples to be collected by an experienced professional [6]. Alternatively, other nasal access swabs have been employed, being more comfortable and with the possibility to be self-collected by the patients. These can be classified into two categories based on their anatomical extent: (i) nasal mid-turbinate swabs (N), where the sampling is executed with patient’s head tilted backwards at 70 degrees, inserting the swab until resistance is met at the turbinate level (2 cm), and rotating the swab several times; and (ii) anterior nares swab (AN), which is accomplished in a similar manner but at a depth of 1 cm without a head tilt [7]. Further, oropharyngeal swabs (O) have been utilized to identify SARS-CoV-2 during the pandemic [8], considering that they offer the least discomfort, in contrast to nasal-access swabs.

More recently, saliva has been suggested as an alternative sample, being the specimen of choice for high-volume testing programs. The idea of using saliva for COVID-19 diagnostics was further supported by observations that SARS-CoV-2 has high binding affinity to angiotensin-converting enzyme (ACE)-2 receptors, which have been found to be highly expressed in the oral mucosa and salivary glands [9,10]. In addition, saliva can be self-collected by the patient, thereby reducing the risk of transmission to medical personnel.

Despite the increasing use of different sampling procedures, there is no consensus on the best collection approach, especially with respect to their impact on the sensitivity of the employed RAD system. It is worth mentioning that the current information on the yield of these sampling approaches is largely based on nucleic acid amplification testing (NAAT), rather than on a systematic approach to compare their sensitivity and specificity for SARS-CoV-2 detection at the antigen level. Lastly, given the varied findings in the literature with respect to each sampling method, it can be hypothesized that pooling three sample types (i.e., O, N, and S) in a single swab will provide greater probability and higher sensitivity to detect SARS-CoV-2 when compared to each sampling method alone.

This study aimed to determine the level of agreement of fluorescent immunoassay-based detection of SARS-CoV-2 protein with the standard, nucleic-acid-based RT-PCR using different swab sampling methods, namely O, N, and S, separately and in combination, in hospitalized COVID-19 patients.

## 2. Materials and Methods

The study was approved by the Institutional Review Board at Imam Abdulrahman bin Faisal University (IRB 2020-02-118). The study was conducted in the period between 23 February 2021 and 18 September 2021, and it complies with the guidelines for Strengthening the Reporting of Observational Studies in Epidemiology (STROBE).

In this prospective study, fifty (n = 50) COVID-19-positive patients, admitted to the quarantine ward at King Fahad University Hospital during the COVID-19 outbreak period in the Eastern Province of Saudi Arabia, were included. The medical histories of the patients were obtained, including the initial diagnosis day and the period of infection. All patients had clinical evaluations including all potential symptoms, such as cough, fever, tiredness, and breathing difficulty. Whether the patients had a COVID-19 treatment protocol or not, all were reported. Further, a total of twenty-six (n = 26) control, COVID-19-negative patients, admitted to King Fahad University Hospital for elective procedures, were asked to participate in the study and included upon acceptance. The control group were all symptom-free at the time of inclusion and analysis. All patients in the test and control groups were confirmed COVID-19 positive and negative, respectively, using nasopharyngeal swabs and a real-time reverse-transcriptase polymerase chain reaction (rRT-PCR) (GeneXpert GX-XVI instrument; Cepheid, Sunnyvale, CA, USA). The rRT-PCR system was used with a Cepheid Xpert Xpress SARS-CoV-2 gene kit, as a standardized procedure for qualitative detection of the envelope (E) and nucleocapsid (N2) genes of SARS-CoV-2. The kit contains RNA extraction and reverse transcription reagents, as well as the primers targeting the E and N2 gene fragments. The kit was used according to the manufacturer’s protocol. Every patient in the test and control groups signed an informed patient consent. The demographic data for all patients are provided in Table 1.

### 2.1. Sample Collection

All patients were instructed not to eat or brush their teeth 1 h before sampling, and every patient was sampled under the standard-of-care protocol of the hospital. Samples were collected from every patient in the test and control groups as follows:

1. Nasal swab alone (N): A 1st flocked swab was used for nasal sampling, according to the instructions for use from the manufacturer of the detection system. The swab was gently passed into one nostril and pushed until resistance was met at the level of the turbinate (less than one inch into the nostril). The swab was rotated 3 times against the nasal wall and then removed.
2. Oropharyngeal swab alone (O): A 2nd flocked swab was gently rotated 3 times against the posterolateral wall of the oropharynx, while avoiding contact with the tongue and other areas of the oral cavity, and then removed.
3. Saliva swab alone (S): A 3rd flocked swab was used to collect saliva. The patient was asked to spit into a sterile plastic cup where the swab was dipped and rotated 3 times and then removed.
4. Combined swab (O/N/S): A 4th flocked swab was used to sample all three compartments (O, N, and S) at once in the following order: The swab was firstly rotated 3 times on the posterolateral wall of the oropharynx, then entered and rotated 3 times in the second nostril, and lastly dipped in saliva spit into a sterile plastic cup.

### 2.2. Sample Analysis

All swab samples were analyzed immediately, at the same site, after each collection using a Sofia 2 Flu + SARS Antigen Fluorescent Immunoassay (FIA) kit and analyzer (Quidel Co., San Diego, CA, USA) according to the manufacturer’s protocol. In brief, the Sofia 2 Flu + SARS Antigen FIA system works on the principle of immunofluorescence, in a sandwiching design, for simultaneous detection and differentiation of the nucleocapsid antigens from SARS-CoV-2, influenza A, and influenza B. The kit includes detection and control components: (i) monoclonal anti-SARS, anti-influenza A, and anti-influenza B antibodies; (ii) a Flu + SARS positive control swab coated with non-infectious recombinant influenza A, influenza B, and SARS antigens; and (iii) a negative control swab coated with non-infectious Streptococcus C antigen. For each sample type (O, N, S, or O/N/S), the swab was immediately placed in the disrupting reagent tube, in which the virus particles were disrupted, exposing internal viral nucleoproteins. Thereafter, the sample was dispensed into the test cassette well. Subsequently, the analyzer injected the sample through a test strip containing the chemical detection environment. If the SARS-CoV-2 viral antigen was present, it was trapped and accumulated in the strip. The analyzer then scanned the test strip and measured the fluorescent signal using a specific algorithm. The analyzer displayed the result as positive, negative, or invalid on the screen. The instrument was calibrated and tested with positive and negative controls after each lot of 25 tests, according to the manufacturer’s instructions.

### 2.3. Statistics

Patient demographics (age, gender, citizenship, civil status), COVID-19 vaccination status, and the period between the antigenic and rRT-PCR tests were all evaluated by examining descriptive statistics. The data are presented as means with standard deviations, medians with ranges, numbers of observations, and/or frequencies in Table 1. Further, the types and frequencies of medications taken during the period of COVID-19 infection and all potential symptoms are presented. The demographic/descriptive variables were compared at the 95% confidence interval using a chi-squared test or the Fisher test. For the primary outcome analysis, Cohen’s kappa coefficient was used to measure the agreement between the previously confirmed rRT-PCR-based detection of SARS-CoV-2 and the detection of SARS-CoV-2 in any of the sample types (O, N, S, or combined O/N/S). Further, Cohen’s kappa test was evaluated with respect to the level of agreement between rRT-PCR results and the detection of SARS-CoV-2 in any of “O”, “N”, or “S” (i.e., if SARS-CoV-2 antigen was detected in any of “O”, “N”, or “S”, then it was considered a positive case). As a secondary outcome measure, correlation analysis was performed between positive SARS-CoV-2 detection in the different samples and the different demographic, medication, and symptom parameters after grouping of the individual symptoms and medications into their relevant respective categories. The statistical analysis was executed using SPSS^®^ Statistics software, Version 25.

## 3. Results

In the total of 50 COVID-19 and 26 healthy control subjects, the median ages were 48.6 (±12.2) and 34.5 (±15.6) years, respectively; these were not significantly different (*p* = 0.06) (Table 1). Similarly, no significant difference was found between the two groups with respect to civil status (Table 1). Regarding gender, more male subjects (94%) represented the COVID-19 group, in contrast to 62% in the control group (*p* < 0.05). Further, although equal numbers of Saudi and non-Saudi (1:1) subjects were included in the COVID-19 group, the control group comprised more Saudi (81%) subjects (Table 1). With respect to vaccination status, where only 2 out of 50 test subjects (4%) had received the first dose of the vaccine, all subjects in the control group had received a COVID-19 vaccine, of which 15 subjects (58%) had received two doses and 26 subjects (100%) had received their first dose of the vaccine. 

### 3.1. Cohn’s Agreement Analysis

The antigenic detection of SARS-CoV-2 revealed significant agreement with the rRT-PCR, using all sampling approaches (O, N, S, and combined O/N/S) (Table 2). All 26 control subjects, diagnosed as COVID-19 negative via rRT-PCR, were found to be negative using the fluorescent immunoassay for all sampling methods (i.e., 100% specificity). On the contrary, the level of agreement with respect to sensitivity varied among the different sampling methods. The lowest sensitivity was recorded for the saliva (S) method (66%; 33 positive detections out of 50 in the COVID-19 rRT-PCR-confirmed group), providing a weak agreement with rRT-PCR with a Cohn’s Kappa coefficient of 0.57. Moderate agreement was confirmed for the rapid antigenic detection with rRT-PCR when using an oropharyngeal (O) swab (Kappa = 0.66; 74% sensitivity) or a nasal (N) swab (Kappa = 0.68; 76% sensitivity). The highest agreement among the four sampling methods was found for the combined O-N-S swab, demonstrating a strong level of agreement with Kappa = 0.8 and 86% sensitivity (Table 2). When considering positive detection with either an O or N swab, as well as with any of the O, N, or S swabs, perfect agreements with rRT-PCR diagnosis were reached, with up to 94% (Kappa = 0.91) and 98% (Kappa = 0.97) sensitivity achieved, respectively (Table 2). 

### 3.2. COVID-19 Symptom Frequencies

**Figure 1:**
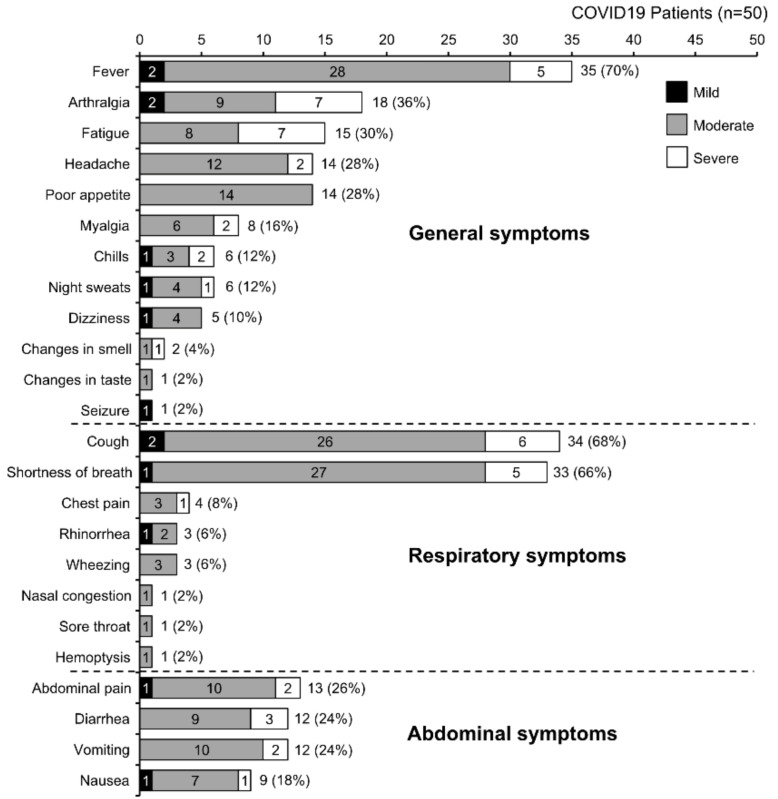
Symptom frequency among the patients in the COVID-19 group.

Figure 1 shows the frequencies of the symptoms in the COVID-19 group. The most frequent symptoms were fever (70%), cough (68%), and shortness of breath (66%). The second level of symptoms comprised arthralgia, fatigue, and headache (28–30%). Abdominal and digestive system symptoms (poor appetite, abdominal pain, diarrhea, nausea, and vomiting) clustered in the third position, ranging from 18% (with nausea) up to 28% (with poor appetite). The least reported symptoms at the time of sampling were sore throat, nasal congestion, change in taste, seizure, and hemoptysis, with each observed in only 2% of the study population.

### 3.3. Medications 

The antiviral medication Favipiravir was prescribed in 30% of the COVID-19 cases. The most prescribed medication was the anticoagulant medication Clexane, prescribed to 72% of the COVID-19 patients, followed by a combination of different vitamins (64%) and the steroidal medication dexamethasone (50%). The antibiotic Ceftriaxone was prescribed for 32% of cases, whereas Azithromycin was prescribed to 26%. All prescribed medications and their frequencies of use during COVID-19 infection are presented in Table 3.

### 3.4. Correlation Analyses

The results of immunofluorescent antigenic detection in the three different swab types (O, N, and S) were correlated with the different independent variables in this study (Table 4). Among all variables entered in the bivariate Pearson correlation, only the age variable demonstrated a significant negative association with viral antigenic detection in the saliva swab (r = −0.65; *p* < 0.001) (Table 4).

## 4. Discussion

The present study demonstrates a high sensitivity (up to 86%) and perfect specificity (100%) for the employed rapid SARS-CoV-2 antigenic detection system from Quidel, particularly when using combined sampling of the oropharynx, nose, and saliva with a single flocked swab. It was also found that although the recommended sample type for this system (nasal swab alone) provided a high sensitivity (76%), this did not reach the manufacturer specifications, reported to be 93%, despite the adherence to the instructions of use for this system. While the discrepancy between these two figures seems to be high, such variability is also evident in the published studies on this RAD system. For example, while sensitivity values of 93.8% [11] and 80.4% [12] have been reported with the Quidel RAD system using a banked combined NP/O swab or NP swab alone, respectively, other studies reported lower sensitivity values of 77% [13] and 72% [14] using fresh NP or N swabs, respectively, immediately analyzed after sampling. In fact, a recent systematic review and meta-analysis reported a pooled sensitivity for the present Quidel RAD system of 77.4%, slightly above the present value. Importantly, the present results highlight the need for testing and employing an appropriate sampling approach for high RAD validity and robustness, considering its prized advantages for easy, rapid, and large-scale screening. 

During the last few years, several studies explored possible sampling alternatives to reduce patient discomfort and eliminate the need for trained personnel, which both represent major drawbacks of nasopharyngeal (NP) swabs. Nonetheless, most of these studies were conducted with respect to the detection of the SARS-CoV-2 RNA using RT-PCR molecular methods. For instance, three independent studies revealed that the combined oropharyngeal/nasal (O/N) swab and the NP swab alone provide similar sensitivity [15,16,17]. These findings were further supported by two independent meta-analyses, revealing equivalent sensitivity of the combined O/N swab and NP (95–97%), but when using these swabs for RT-PCR molecular detection [18,19].

In contrast, fewer studies have tested this combined swab (i.e., O/N) as a comfortable swab sample for RAD and compared it against NP swabs for RT-PCR. In one study, the use of a combined O/N swab for three selected lateral-flow-based RAD systems provided relatively good sensitivities of 78.2% (Innova system), 74.4% (Encode system), and 60.3% (SureScreen system), as contrasted with RT-PCR [20]. Nonetheless, it was not clear in the latter study whether the reference RT-PCR detection was conducted using the NP swab or, as in the test groups, using a combined O/N swab. On the contrary, a study by Agulló and co-workers employed nasal and saliva combined sampling with a single swab for another RAD system (Panbio system) and revealed a rather low sensitivity of 49.6% when contrasted with NP swabs and RT-PCR detection [21]. Collectively, although there are some variations among the studies, the findings of the present and the previous studies suggest that the use of triple-component combined swabs (i.e., combined O/N/S swabs) further improves the RAD sensitivity compared to when only two components (O/N or S/N) are used.

During the pandemic years, high expectations have been raised to use saliva as a comfortable sample that would eventually be collected by the patients themselves, providing easier procedures and less risk of infection spread. Nevertheless, varied results have been so far obtained with respect to the range of sensitivity when using saliva samples alone. Further, these diverse findings have been reported not only for RAD but also for the highly sensitive RT-PCR methods. For instance, Barat and co-workers found the positive (sensitivity) and negative (specificity) percentages of agreement of saliva samples to be 81.1% and 99.8%, respectively, when evaluated against NP swabs for RT-PCR detection [22]. In the same manner, Fronza and co-workers compared saliva samples vs. NP swabs for molecular RT-PCR detection; however, relatively lower sensitivity (68.9%) and specificity (88.6%) values were observed in the latter study [23] as compared to the former one [22]. On the other hand, a generally much lower range of sensitivity (8.1–55.6%) has been registered for the saliva type of samples when used with different types of RAD systems, despite the excellent recorded specificities [24,25,26,27]. The present comparative study conforms with the previous studies, showing the least sensitivity value for the saliva alone among all evaluated sample types. 

Several factors have been suggested to contribute to the relatively high false-negative results of RAD systems, including the viral load, time of testing since the start of symptoms, severity of symptoms, type of sample (whether fresh or banked), material of the swab, and so on. In addition to lacking the viral load parameter, one potential factor for the relatively high false-negative results is the period between the onset of symptoms and the analysis in this study, which ranged between 0 and 14 days with an average of 5.6 days; this is slightly above the manufacturer recommendation (within the first 5 days of the onset of symptoms). Nonetheless, the present correlation analysis did not find significant associations between such parameters and the sensitivity of the different samples, except for the negative correlation between the patient age and the RAD sensitivity when using the saliva sample alone. The exact mechanisms behind this finding are not yet known, but, speculatively, age-related changes in saliva properties (quantity, viscosity, and content) may have contributed.

We also calculated the sensitivity of the present RAD system where positive detection was granted when viral particles were detected in any of the three analyzed samples (either O, or N, or S; providing 98% sensitivity) or two of the analyzed samples (either O or N; providing 94% sensitivity). The hypothesis here is that in each patient, if the virus is not detected in one sample type, it will likely be detected in another sample type. Nevertheless, a larger cohort is warranted to prove such an observation.

A limitation in this study was that the viral load was not measured in this cohort of patients. This would have provided additional information on whether the viral load had influenced the sensitivities of the different sampling methods. Another limitation is that mainly male patients (94%) were coincidentally available and agreed to be enrolled in the study, which may result in skewness of the results towards a single gender.

## 5. Conclusions

Efficient testing methods are a vital tool to arrest the spread of SARS-CoV-2, especially for large-scale screening programs. The emergence of new variants of SARS-CoV-2 has generated more uncertainty in suitable testing methods, leading to inconsistency in testing techniques that risks the efficacy of validated tests and sample collection methods. Our study results depict that rapid antigenic detection of SARS-CoV-2 nucleocapsid using a fluorescent immunoassay system conveys reliable diagnostic yield with the combined O/N/S sampling method using a single swab, providing the highest sensitivity, while maintaining perfect specificity. In contrast, lower sensitivities were associated with the application of oropharyngeal (O) and nasal (N) swabs, and even worse sensitivities were associated with the saliva swab alone, despite all presenting perfect specificities. Future studies with a larger sample size should examine the safety and efficacy of using combined sampling approaches for rapid SARS-CoV-2 antigen detection.

## Figures and Tables

**Table 1:** Demographic description. Descriptive data show the patient demographics in the test (COVID-19 positive) and control (healthy) groups as confirmed via rRT-PCR testing.

**Continuous Variables; Mean [STDEV; Median] (Range)**		**COVID-19 (n = 50)**	**Control (n = 26)**
Age (Years)		48.6 [12.2; 50] (23–69)	34.5 [15.6; 32] (14–83)
Time between onset of symptoms and RAD (Days)		5.60 [2.62; 5] (0–14)	n.a.
Time between rRT-PCR and RAD (Days)		2.8 [2.0; 2] (0–7) *	0.35 [1.06; 0] (0–5) *
**Nominal variables; n (%)**		**COVID-19 (n = 50)**	**Control (n = 26)**
Gender	Female	3 (6%) *	10 (38%) *
	Male	47 (94%) *	16 (62%) *
Citizenship	Saudi	25 (50%)	21 (81%)
	Other nationalities	25 (50%)	5 (19%)
Civil status	Single	16 (32%)	12 (46%)
	Married	34 (68%)	14 (54%)
COVID-19 vaccination	1st dose	2 (4%)	26 (100%)
	2nd dose	0 (0%)	15 (58%)

* *p* < 0.05.

**Table 2:** The table shows the level of agreement (Cohen’s kappa coefficient), sensitivity, and specificity of COVID-19 antigen testing using different sampling methods in relation to rRT-PCR. The evaluated sampling methods were nasal (N), oropharyngeal (O), extraoral saliva (S), and combined (O/N/S) swabs. Further, the results of the two separate methods (N) and (O), and those of the three separate methods (N), (O), and (S), were evaluated.

	Number of Swabs	+VE Result in COVID-19 Group	+VE Result in Control Group	Kappa Coefficient	Sensitivity	Specificity
Oropharyngeal (O)	1	37/50	0/26	0.66 *	74%	100%
Nasal (N)	1	38/50	0/26	0.68 *	76%	100%
Saliva (S)	1	33/50	0/26	0.57 *	66%	100%
Combined (O/N/S)	1	43/50	0/26	0.80 *	86%	100%
Sum of (O) and (N)	2	47/50	0/26	0.91 *	94%	100%
Sum (O), (N), and (S)	3	49/50	0/26	0.97 *	98%	100%

* *p* < 0.05.

**Table 3:** Medications administered during COVID-19 infection.

	Drug	Number of Patients	Percentage
Antiviral medication	Favipiravir	15	30%
Vitamins	Vit C	32	64%
	Vit B1/B6/B12	32	64%
	Vit D	31	62%
	Multi-vitamin	9	18%
Proton pump inhibitors	Nexium	15	30%
	Pantoprazole	24	48%
Antibiotics	Ceftriaxone	16	32%
	Azithromycin	13	26%
Anticoagulant	Clexane	36	72%
Steriods	Dexamethasone	25	50%
Antidiabetic	Insulin	21	42%
Acetaminophen	Paracetamol	15	30%
Calcium channel blocker	Amlor	10	20%
Diuretic	Lasix	10	20%
Laxative	Movicol	6	12%

**Table 4:** Correlation analysis. The table shows all study variables entered in the Pearson bivariate correlation matrix with respect to positive antigen detection using the different sampling methods in the COVID-19-confirmed patients (n = 50). The statistically significant correlation is presented with its correlation coefficient (r) and level of significance (*p*-value).

Variables		Antigen Detection in Oropharyngeal Samples	Antigen Detection in Nasal Samples	Antigen Detection in Saliva Samples
Patient-related factors	Gender, nationality, civil status, age	None	None	Age (−0.65; <0.0001)
Test-related factors	Time between onset of symptoms and RAD (Days)	None	None	None
	Time between rRT-PCR and RAD (Days)	None	None	None
Medications	Antiviral medication	None	None	None
	Vitamins	None	None	None
	Proton pump inhibitors	None	None	None
	Antibiotics	None	None	None
	Anticoagulant	None	None	None
	Steriods	None	None	None
	Antidiabetic	None	None	None
	Acetaminophen	None	None	None
	Calcium channel blocker	None	None	None
	Diuretic	None	None	None
	Laxative	None	None	None
Symptoms	General symptoms	None	None	None
	Respiratory symptoms	None	None	None
	Abdominal symptoms	None	None	None

## Data Availability

The data presented in this study can be requested from the corresponding author.
